# Round the Hospitals

**Published:** 1919-01-04

**Authors:** 


### ROUND THE HOSPITALS.

"Ierne's" article on page 274 of last week's The Hospital, entitled "What Nurses Expect and Want," should be read and studied by every member of the Council of the College of Nursing. "Ierne," with that directness and fearlessness which have won such general acceptance and much appreciation from our readers, entitled this contribution "Wake up, College of Nursing." We have received an urgent request for a large number of copies of last week's issue, which we regret it has been impossible to supply owing to it being sold out. We could wish that every member of the College Council would make it their business to obtain and file The Hospital, following the practice of many hospitals throughout the country. They would then have at hand for reference, as so many hospital superintendents have, material which is up to date and of real value for constant reference. A very ardent supporter of the College, and a zealous worker in its service, like an increasing number of our correspondents, maintains with truth, that the time is approaching, if it has not already come, when every member of the College Council must be prepared to give the necessary time and energy to the important and extending duties which it is their privilege to discharge with the maximum of ability which each may possess. They will find certain important aspects of registration and the College responsibilities dealt with in the "Outlook" in the *Nursing Mirror* this week, page 203.

It may be interesting to note that, whereas the College of Nursing is successfully endeavouring to build up a great National Fund for Nurses, the editress of the publication which devotes so much of its space to denunciations of the College and all its

**ROUND THE HOSPITALS—(*continued*).**

works, concludes her latest annual fairy tale of "Principal Nursing Events" with the words: "We shall continue to claim for nurses the right of self-determination, self-support, and self-expression." It will be news to British nurses that they have ever ceased to have "the right of self-determination, self-support, and self-expression." The best answer trained nurses can give to the party of tarradilloes and make-believe is at once to register their names with the College of Nursing or, being already members of the College, that they should devote as much time as possible during the next three months to secure that the number of trained nurses on the College Register shall, with as little delay as possible, be increased to 20,000 at least. If trained nurses accomplish this they will demonstrably prove the undoubted fact that the right of self-determination, self-support, and self-expression, in the case of every nurse, is a living force which she values and intends to use, in her own interests and defence, when opportunity offers. Meanwhile, members of the College should enter in their diaries under Thursday, January 23, 1919, an evening meeting at which the Bill promoted by the College of Nursing for the State Registration of Nurses will be fully explained. Major Chapple, who had charge of the Fenwick Registration Bill, was at the bottom of the poll in the Stirling and Clackmannan election. There were three candidates.

---

Within a few days of each other Edinburgh and Glasgow have opened nurses' clubs worthy in all respects of the profession they are intended to serve. The King George and Queen Mary Club at Edinburgh for nurses from the overseas Dominions and America was started experimentally some month or two ago in Rutland Square, under the presidency of Lady Linlithgow, and has already proved such an overwhelming success that it is now installed in fine central premises at 27 Drumsheugh Gardens. It owes its foundation to the initiation of the Victoria League, whose president, the Dowager Countess of Jersey, was present at the opening.

---

In Glasgow the Scottish Nurses' Club at 205 Bath Street was opened by the Lord Provost, with the Marchioness of Ailsa, President of the Club, in the chair. She was supported by Mrs. Strong, President of the Scottish Nurses' Association, Miss Finn, matron of the Abbey Hospital, Paisley, who has done much in common with her nurses to promote the interests of the club, and a large company of sisters and nurses. The membership is already 450, and the Lord Provost remarked, in anticipating an early enlargement, that "the generosity of the citizens of Glasgow will not be wanting to help." Thanks to generous help already given, the club starts entirely free of debt.

---

The College of Nursing has a strong centre at Edinburgh under the presidency of Miss Gill, R.R.C., and has just completed a course of lectures presenting exceptional interest from the professional point of view, in conjunction with the National Council for Combating Venereal Diseases. The centre is thoroughly alive and keen in the prosecution of research into the causes and remedies of social evils. The Glasgow Centre, formed last November under the joint presidency of Miss Melrose, R.R.C., Royal Infirmary, Glasgow, and Miss Gregory Smith, R.R.C., Western Infirmary, Glasgow, has already enlisted widespread interest and local support.

---

The nurses' bazaar in aid of the formation of a centre for the College of Nursing in Leeds, as well as for the Nation's Fund for Nurses, was held in the Victoria Hall in that town, and was a very brilliant and picturesque affair. The stalls were grouped to represent a market-place, and on one was shown some interesting specimens of hand-loom weaving done by disabled soldiers. A wealth of needlework, as well as some charming china and trifles which it would be invidious perhaps to describe as "very suitable for Christmas presents," tempted a constant stream of purchasers, and an excellent orchestra of wounded soldiers added to the gaiety of the scene. The proceeds, we learn, were £3,000.

---

Already institutions urgently needed by the civil population are resuming their normal activities after a long interval devoted to the care of sick and wounded soldiers. The children's ward in the Boscombe Branch of the Royal Victoria and West Hants Hospital is one of the departments recently reopened, and very urgently required by the neighbourhood it serves. It proved to need a complete new equipment to refit it for the use of the young; and the Linen Association, which is exceptionally well organised at Bournemouth, raised the required sum, £70, with the least possible delay. Members of the Linen Association this year have contributed 2,150 articles for use in the Royal Victoria Hospital, and have sent in £150 in money for the purchase of blankets, quilts, and special articles.

---

At Bermondsey Poor-Law Infirmary the probationers appear to be in a state of seething discontent, which has manifested itself in that ugliest of forms, a refusal to go on duty until grievances are redressed. The medical superintendent of the infirmary stated the facts to the Bermondsey Board of Guardians, and explained that the main cause of unrest was the suspension of the whole day weekly leave on account of shortage of staff, and the omission of the Guardians to grant any war bonus to the nursing staff. The board at once undertook to restore the whole day, a privilege which, once conceded, must, we can well understand, be deemed indispensable, and are "considering" the redressal of other grievances. The system of totally ignoring the just requirements of the staff until they are forced to notice an outbreak of indiscipline is one which we have often had occasion to condemn. It cuts at the root of all decent feeling between the authorities and the staff, exalts rebellion into a virtue, and penalises the women who suffer in silence rather than adopt a truculent attitude unworthy of their profession. The

**ROUND THE HOSPITALS**—*(continued)*.

insufficiency of the powers granted to the matrons of infirmaries by Boards of Guardians is often the root cause of these regrettable occurrences.

---

Lord Glenarthur, Chairman of the Western Infirmary, Glasgow, made a serious statement at the annual Christmas meeting which took place at the hospital on December 23. He explained that the average daily number of in-patients during the year had been 610, a record in its history, and that the strain on the matron, Miss Gregory Smith, and her nurses had been unusually great. The managers were most anxious to reduce the hours worked by the nurses, but found themselves impeded by lack of bedroom accommodation for the extra nurses such a reform would entail. He appealed for funds to build a considerable extension to the nurses' home. We trust Glasgow will not long permit itself to lie under the stigma of over-working the nurses in this famous infirmary. The demand for shorter working hours for nurses has the backing of an influential public, and no institution can afford to deny it, or long delay to answer its call.

---

The continuation of Sir Douglas Haig's despatch of November 8 submitting names deserving of special mention includes a very large number of members of Q.A.I.M.N.S., the Reserve, T.F.N.S.; the V.A.D.; Special Probationer Nursing Service, the British Red Cross Society and both trained and voluntary workers. Names of distinguished members of the Canadian, Australian, New Zealand and South African Army Nursing Services are also published. Members of the Army Nursing Corps of the American Expeditionary Force have also been singled out for special mention.

---

The King held an Investiture in the Ball-room of Buckingham Palace on December 21, at 10.30 a.m. Among the decorations personally conferred by the King was that of the Royal Red Cross, First Class, to Matron Katharine Hagar, American Nursing Service (Harvard Unit). The following were admitted by His Majesty to be Associates of the Order of the Royal Red Cross:—Matron Florence Tosh and Assistant Matron Rose Rorke, Q.A.I.M.N.S.; Sister Isabel Anderson, Sister Jennie Cameron, and Sister Mary Dunbar, of the Reserve; Sister Alice Lowe and Sister Maud Taylor, T.F.N.S.; Matron Emily Porter, C.N.S.; Miss Ruth Lindsay, Miss Annie Philip, and the Lady Tollemache, V.A.D.; Sister Helen Hinckley, American N.S. The King bestowed the Military Medal for bravery in the field on Sister Linda Bowles, Q.A.I.M.N.S. (R.). The band of the Grenadier Guards played during the investiture. At its conclusion Queen Alexandra received at Marlborough House the members of the Military Nursing Service whose names have been mentioned above.

---

The New Year Honours include the following awards in recognition of valuable services in connection with the war: Bar to Royal Red Cross, First Class, Miss Annie Warren Gill, Lady Superintendent of Nurses, Royal Infirmary, Edinburgh. Royal Red Cross, Second Class, *Q.A.R.N.N.S. Reserve, Nursing Sisters* Dora Lund, Nance McKay, Mabel Rose Chester-Webb, Margaret Hunt, Lilian Swift, Louisa C. Chamberlain; and to the following: The Misses Mary Edgar, head sister, London Homœopathic Hospital, Great Ormond Street, W.C.; Ina Docherty, matron, General Hospital, Great Yarmouth; Elizabeth Wright Jones, matron, Monkstown Hospital, Kingstown; Edwardes, matron, R.N. Convalescent Hospital, Great Malvern; Jessie Annie Mortlock, matron, Brooksby Hospital, Leicestershire. The following appointments to the Order of the British Empire for valuable services rendered in connection with military operations in France and Flanders are also made under date of January 1: *C.B.E. (Military Division)*—Miss E. B. Ridley, R.R.C., principal matron, Canadian Nursing Service; Miss G. M. Wilson, R.R.C., principal matron, Australian Army Nursing Service; *O.B.E. (Military Division)*—Miss B. J. Willoughby, R.R.C., matron, Canadian Army Medical Corps.

---

The new Army Council Instruction substituting increased rates of uniform allowance to Army nurses in place of the former allowance would have been more welcome had it been issued three months ago. Members of Q.A.I.M.N.S., the Reserve, and the T.F.N.S. are, for the future, to be allowed £10 on enrolment to cover the cost of a winter outfit. For the summer outfit further instructions are promised. V.A.D. nursing members and military probationers are to be allowed £4 each six months, payable in advance, £2 of which will be claimed as a refund in case they cease to serve within three months without due cause. It is stated that these increases are purely temporary. But the uniform allowance for army sisters has always been based on a rather "near" estimate of rigid necessaries; it had always to be supplemented out of the wearer's pocket, and we hope it will never be reduced to its former level.

---

We have more than once expressed the opinion that the zeal shown by the public in Red Cross working parties and supply depôts ought not to be allowed to die down after the war, but should be turned in the direction of supporting voluntary hospitals. We are glad to see that Mrs. Albert Barrett, at the Palmer's Green sale for the Red Cross before Christmas, entreated those who had contributed the work to "switch their efforts on to the Great Northern Hospital" after peace is signed. The hospital serves a wide district, part of which is very poor, and would be greatly strengthened by liberal supplies of material and such regular contributions in money as could be furnished if working parties were re-organised throughout the borough for this purpose.

---

A pleasant little function took place at Rye on Christmas Eve at the House of Mrs. Dacre Vincent, commandant of Mrs. Jameson's military hospital,

**ROUND THE HOSPITALS—(*continued*).**

who entertained at tea the matron, Miss Lyon, and all the voluntary workers, and presented each with a token of remembrance. Miss Lyon was presented with a very handsome set of golf clubs and bag, the gift of Rye voluntary workers who have enjoyed the privilege of helping at the hospital during her term of office, which began in March 1916. On Christmas morning Miss Lyon received another token of the affection and esteem she has roused among her workers in a silver cream-jug and sugar-basin from the hospital voluntary workers in Winchester.

The American Unit of the Scottish Women's Hospitals has been doing amazingly fine work at Vranja in the heart of Serbia. The cold is intense and the ravages wrought by influenza overwhelming. They found the hospital in a terrible state and were faced with prodigious difficulties in getting the wards cleaned up, as they possessed not a single brush, duster, pail, or piece of soap. They finally did their cleaning operations with the aid of brushes made from the branches of trees round the hospital. They report that the suffering and sadness all round are heart-rending. A typical patient was a Serbian officer who had returned after three years' separation, full of joy and hope, only to find his house burned to the ground and his wife and little children hanged by the Bulgars. Many of the patients are English soldiers and of them a happy description is given: "When I feel extra tired," says a member of the unit, "I go into the English Tommies' ward and it revives me to see them looking so comfortable and so appreciative."

On Christmas morning Miss Tait McKay, matron of the 4th Southern General Hospital, Plymouth, received a surprise present, accompanied by the best wishes of the medical officers, the nursing, general service and labour staffs, N.C.O.s and men of the Salisbury Road and Hyde Park Hospitals. The gift, which was placed on her breakfast-table, consisted of a beautiful silver (Chippendale) tea-service, comprising teapot, cream-jug and sugar-basin, with a handsome silver hot-water jug, together with a Doulton rose-bowl.

